# Relationship between simultaneous exposure to ergonomic risk factors and work-related lower back pain: a cross-sectional study based on the fourth Korean working conditions survey

**DOI:** 10.1186/s40557-018-0269-1

**Published:** 2018-09-05

**Authors:** Jae-Yeop Kim, Ji-Su Shin, Myeong-Seob Lim, Hyeon-Gyeong Choi, Sung-Kyeong Kim, Hee-Tae Kang, Sang-Baek Koh, Sung-Soo Oh

**Affiliations:** Department of Occupational and Environmental Medicine, Wonju Severance Christian Hospital, Wonju College of Medicine, Yonsei University, Wonju, South Korea

**Keywords:** Work-related low back pain, Ergonomic risk factors, Simultaneous exposure, Fourth Korean working conditions survey (KWCS)

## Abstract

**Background:**

It is well known that ergonomic risk factors and back pain are related. However, few studies have examined the relationship between simultaneous exposure to these risk factors and back pain in a Korean population. We aimed to investigate the relationship between simultaneous exposure to ergonomic risk factors and work-related lower back pain (LBP) based on the fourth Korean Working Conditions Survey (KWCS).

**Method:**

The fourth KWCS (2014) was used for this study. Chi-square tests and logistic regression were used to assess relationship between 5 ergonomic risk factors and work-related LBP. We also analyzed the relationship between simultaneous exposure to 2 risk factors and work-related LBP.

**Results:**

All 5 ergonomic risk factors (fatigue-inducing and painful posture; lifting or moving people; dragging, pushing, or moving heavy objects; standing posture; and repetitive hand or arm movements) were significantly correlated with work-related LBP in the severe exposure group (adjusted odd ratios [aOR] 5.09, 95% confidence interval [CI] 4.46–5.83; aOR 1.98, 95% CI 1.62–2.42; aOR 2.09, 95% CI 1.82–2.40; aOR 1.79, 95% CI 1.60–2.01; aOR 2.04, 95% CI 1.82–2.30, respectively). When exposed to 2 risk factors simultaneously, the relationship between exposure and work-related LBP was not greater than exposure to only 1 risk factor in our study (usually exposed to ‘fatigue-inducing or painful posture’ aOR 2.17, 95% CI 2.02–2.34; high exposure to both ‘fatigue-inducing or painful posture’ and ‘dragging, pushing, or moving heavy objects’ aOR 2.00, 95% CI 1.82–2.20).

**Conclusions:**

There was a strong relationship between severe exposure to each ergonomic risk factor and work-related LBP. However, when exposed to 2 ergonomic risk factors simultaneously, the relationship between exposure and work-related LBP was not stronger than when exposed to only 1 risk factor in our study.

## Background

Work-related musculoskeletal disorder (WMSD) is an important health issue worldwide. In multiple countries, musculoskeletal disorders account for the majority of work-related disorders [1, 2], and it is well documented that WMSD incurs substantial social costs [3, 4]. In South Korea, WMSD accounted for 65.8% of all work-related disorders in 2016 [5].

Multiple studies have reported that exposure to ergonomic risk factors induces or worsens musculoskeletal disorders [6–8]. Fatigue-inducing or painful posture, repetitive hand or arm movements, prolonged standing or sitting, and inflicting excessive force are examples of ergonomic risk factors [9]. WMSD develops over time, and do not occur as a result of sudden or temporary events [10]. There have been several studies investigating possible approaches to reducing exposure to these ergonomic factors [11–13]. WMSD has been a topic of considerable research interest in Korea since the 1960s [14], with the majority of studies conducted within occupation-specific workplaces [15–19].

It is well known that ergonomic risk factors showed a significant association with LBP [20, 21]. In previous epidemiological studies, back pain has been known to have a strong association with lifting, forceful movement and wholebody vibrations, and a significant association with awkward posture and heavy physical work. In the 1997, the report of the National Institute for Occupational Safety and Health of USA have shown that there were few combined effects of these risk factors on LBP [22]. However, several studies suggested that both lifting and awkward postures were important contributors to the risk of LBP [22]. It is expected that a risk factor (e.g., forceful exertion) in the presence of another risk factor (e.g., repetitive work) or factors (e.g., high repetition in an awkward posture) will interact resulting on WMSD precipitation [9], even though definite multipliers for the interactions still needs to be defined [23]. However, there have been few epidemiological studies investigating relationship between simultaneous exposure to ergonomic risk factors and work-related LBP.

This study analyzed the relationship between ergonomic risk factors and work-related LBP. Particularly, we wanted to investigate the relationship between the simultaneous exposure to these risk factors and LBP, utilizing data from the fourth Korean Working Conditions Survey (KWCS).

## Methods

### Participants

This study utilized data from the fourth KWCS, conducted by the Korea Occupational Safety and Health Agency (KOSHA) in 2014. The KWCS is administered to working individuals aged ≥15 years through household visits and interviews. A total of 50,007 people participated in the fourth KWCS. The reliability and validity of the KWCS has already been established [24].

Individuals with missing values for major variables and covariates were excluded from the analysis. In addition, individuals with responses that were marked, “I don’t know or no answer” or “refuse to answer” were also excluded. Who were soldiers or who had an employment status of “unpaid family work” or “other work” were excluded due to a small number of such cases. Finally, 9255 were excluded from the analysis and a total of 40,752 participants were included in the analysis.

### Major variables

To ensure that only work-related LBP were included, only people who responded with “lower back pain” to the question “Have you had the following health problems in the past 12 months?” and answered “yes” to the additional question “If you had, did it result from your work?” were considered as having work-related LBP. Individuals who responded with “I don’t know/no answer” or “refuse to answer” were excluded from the analysis.

All 5 ergonomic risk factors included in the fourth KWCS survey were included in the analysis. The risk factors were: “fatigue-inducing or painful posture,” “lifting or moving people,” “dragging, pushing, or moving heavy objects,” “standing posture,” and “repetitive hand or arm movements.” There were 7 levels of exposure to each risk factors (no exposure at all, hardly any exposure, ¼ of the working hour, ½ of the working hour, ¾ of the working hour, almost the entire working hour, and the entire working hour). Based on survey results, exposure to risk factors was reclassified into 4 categories: none, mild, moderate, and severe. ‘No exposure at all’ was classified as none, ‘hardly any exposure at all’ and ‘1/4 of the working hour’ were classified as mild exposure, ‘1/2 of the working hour’ and ‘3/4 of the working hour’ considered moderate exposure, and ‘almost the entire working hour’ and ‘the entire working hour’ were classified as severe exposure.

### Covariates

Potential confounding variables included sex, age, occupational class, type of employment, working hours, shift work, number of employees in the workplace, level of education, income, autonomy in taking breaks during work, and vibration exposure. Age was divided into 5 groups: under 30, 30–39 years, 40–49 years, 50–59 years, and above 60 years. Based on the Korean Standard Occupational Classification (6th revision) [25], the KWCS data investigated 10 occupation types, and also surveyed soldiers. Occupation was classified into 3 categories: white collar (managers, professionals, technicians and semi-experts, and office workers), service workers (service workers and sales workers), and blue collar (skilled agricultural and fishery workers, functional operators and relevant functional workers, equipment, machinery handlers and assembly workers, and simple laborers).

Nonpaid family workers and other workers were excluded from the analysis due to small sample size, resulting in 3 types of included employment: self-employed without employees, self-employed with employees, and paid workers. Working hours were classified into 3 groups: under 40 h per week, 40–60 h per week, and greater than 60 h per week. The number of employees in the workplace was divided into 4 groups: under 5, 5–49, 50–299, and more than 300.

Autonomy with taking breaks during work was examined with the question, “Can you take a break when you want?” to which 5 responses were possible: always, most of the time, sometimes, not really, and not at all. These responses were then clustered into 3 categories: “always” and “most of the time” were grouped into “mostly,” “sometimes” and “not really” were grouped into “sometimes,” and “not at all” was grouped into “never.”

Vibration exposure was assessed with the question, “How much are you exposed to hand-transmitted vibration or vibration generated by machinery?” 7 responses were possible: no exposure at all, hardly any exposure, ¼ of the working hour, ½ of the working hour, ¾ of the working hour, nearly the entire working hour, and the entire working hour. These were then clustered into 4 groups, as follows: “No exposure at all” was reclassified as “never,” “hardly any exposure” or “1/4 of the working hour” into “mild,” “1/2 of the working hour” or “3/4 of the working hour” into “moderate,” and “nearly the entire working hour” or “entire working hour” into “severe.”

### Analysis

The chi-square test was used to examine the general characteristics of the study population with regarding to the work-related LBP and trend test was also conducted. Relationship between the 5 ergonomic risk factors and work-related LBP were examined using logistic regression. Analyses were performed after adjusting for sex, age, occupational classification, type of employment, working hours, shift work, number of employees in workplace, education, income, autonomy of taking breaks during work, and vibration exposure.

The relationship between simultaneous exposure to ergonomic risk factors and work-related LBP was examined using logistic regression. Exposure to 2 risk factors was classified into 4 groups according to Table 1.

**Table 1:** Classification for simultaneous exposure to 2 ergonomic risk factors

		Ergonomic risk factors A			
		Never	Mild	Moderate	Severe
Ergonomic risk factors B	Never	1. Little exposure to A, B		2. Usually exposed to A	
	Mild				
	Moderate	3. Usually exposed to B		4. High exposure to A, B	
	Severe				

Compared to group 1, relationships between groups 2, 3, and 4 and lower back symptoms were analyzed with logistic regression (Table 1). Sex, age, occupation, type of employment, working hours, shift work, number of employees in workplace, education, income, autonomy of taking breaks during work, and vibration exposure were adjusted for. After selecting 2 risk factors (A and B in Table 1) to analyze the effects of simultaneous exposure, we also adjusted for the remaining 3 ergonomic risk factors.

The data were analyzed using the IBM SPSS 23.0 software (Chicago, IL, USA).

## Results

The general characteristics of participants are shown in Table 2. From a total of 40,752 participants included in the final analysis, 21,462 were male and 19,290 were female. With the exception of shift work, there were significant differences in the prevalence of work-related LBP across groups for all variables. The prevalence of work-related LBP was higher in self-employed individuals with no employees than in paid workers, and symptoms were found to increase with a decreasing number of employees in the workplace. Work-related LBP were found to increase with higher exposure to each ergonomic risk factors, except ‘lifting or moving people’.

**Table 2:** Demographic and exposure characteristics of the study population

	Work-related LBP		
Total (*n* = 40,752)	Yes (n, %)	No (n, %)	*p*-value
Sex			< 0.001
Male	2815 (13.1)	18,647 (86.9)	
Female	3221 (16.7)	16,069 (83.3)	
Age			< 0.001
< 30	257 (6.2)	3896 (93.8)	
30–39	740 (9.1)	7312 (90.9)	
40–49	1425 (12.8)	9669 (87.2)	
50–59	1513 (16.2)	7804 (83.8)	
≥ 60	2101 (26.0)	5975 (74.0)	
Occupation type			< 0.001
White collar	890 (7.0)	11,819 (93.0)	
Service workers	1862 (13.5)	11,950 (86.5)	
Blue collar	3284 (23.1)	10,947 (76.9)	
Employment status			< 0.001
Self-employed without employees	2510 (23.7)	8103 (76.3)	
Self-employed with employees	312 (13.1)	2077 (86.9)	
Paid workers	3214 (11.6)	24,536 (88.4)	
Working hours (/week)			< 0.001
< 40	1370 (20.7)	5243 (79.3)	
40–59	2953 (12.0)	21,736 (88.0)	
≥ 60	1713 (18.1)	7737 (81.9)	
Shift work			0.578
Yes	445 (14.5)	2634 (85.5)	
No	5591 (14.8)	32,082 (85.2)	
Number of employees			< 0.001
< 5	3619 (19.3)	15,088 (80.7)	
5–49	1656 (11.2)	13,081 (88.8)	
50–299	537 (10.7)	4504 (89.3)	
≥ 300	224 (9.9)	2043 (90.1)	
Education			< 0.001
Below elementary	1348 (32.6)	2786 (67.4)	
Middle & high school	3298 (16.6)	16,537 (83.4)	
Above university	1390 (8.3)	15,393 (91.7)	
Income (10,000₩/month)			< 0.001
< 100	1580 (23.6)	5127 (76.4)	
100–199	2138 (15.8)	11,429 (84.2)	
200–399	1898 (11.6)	14,454 (88.4)	
≥ 400	420 (10.2)	3706 (89.8)	
Rest during work time			< 0.001
Always & most	2250 (16.4)	11,428 (83.6)	
Sometimes	3389 (13.6)	21,611 (86.4)	
Never	397 (19.1)	1677 (80.9)	
Vibration exposure			< 0.001
None	2164 (11.8)	16,180 (88.2)	
Mild	2785 (16.9)	13,653 (83.1)	
Moderate	665 (16.9)	3268 (83.1)	
Severe	422 (20.7)	1615 (79.3)	
Fatiguing or painful posture			< 0.001
None	386 (5.5)	6575 (94.5)	
Mild	2436 (11.8)	18,191 (88.2)	
Moderate	2077 (21.7)	7499 (78.3)	
Severe	1137 (31.7)	2451 (68.3)	
Lifting or moving people			< 0.001
None	3548 (15.1)	20,004 (84.9)	
Mild	2042 (13.8)	12,727 (86.2)	
Moderate	285 (14.9)	1630 (85.1)	
Severe	161 (31.2)	355 (68.8)	
Dragging, pushing, or moving heavy objects			< 0.001
None	986 (8.8)	10,202 (91.2)	
Mild	3334 (15.5)	18,217 (84.5)	
Moderate	1291 (20.6)	4982 (79.4)	
Severe	425 (24.4)	1315 (75.6)	
Standing posture			< 0.001
None	577 (9.2)	5665 (90.8)	
Mild	2108 (12.9)	14,202 (87.1)	
Moderate	2006 (17.1)	9694 (82.9)	
Severe	1345 (20.7)	5155 (79.3)	
Repetitive hand or arm movements			< 0.001
None	448 (8.0)	5148 (92.0)	
Mild	1562 (11.4)	12,187 (88.6)	
Moderate	2061 (17.0)	10,066 (83.0)	
Severe	1965 (21.2)	7315 (78.8)	

Abbreviations: *LBP* lower back pain

Test for trend was conducted for all variable except ‘shift work’

Relationship between the degree of exposure to each risk factor and work-related LBP are shown in Table 3. For ‘fatigue-inducing or painful posture’, ‘dragging, pushing, or moving heavy objects’, ‘standing posture’ and ‘repetitive hand or arm movements’ risk factors, the relationship between exposure and work-related LBP increased with increasing severity of exposure, compared to the no exposure group, regardless of adjustment. For the ‘lifting or moving people’ risk factor, the mild and moderate exposure groups tended to have less LBP than the exposed groups (adjusted OR [aOR] 0.82, 95% confidence interval [CI] 0.77–0.88; aOR 0.78, 95% CI 0.68–0.89, respectively). However, the severe exposure group showed a correlation with work-related LBP (aOR 1.98, 95% CI 1.62–2.42).

**Table 3:** Logistic regression analysis for ergonomic risk-factor exposure and work-related LBP

	Work-related LBP	
Ergonomic risk factor	Crude	Adjusted^a^
Fatigue-inducing or painful posture		
None	(ref)	(ref)
Mild	2.28 (2.04–2.54)*	1.86 (1.65–2.08)*
Moderate	4.71 (4.21–5.28)*	3.27 (2.89–3.69)*
Severe	7.90 (6.97–8.94)*	5.09 (4.46–5.83)*
Lifting or moving people		
None	(ref)	(ref)
Mild	0.90 (0.85–0.95)**	0.82 (0.77–0.88)*
Moderate	0.98 (0.86–1.12)	0.78 (0.68–0.89)*
Severe	2.55 (2.11–3.09)*	1.98 (1.62–2.42)*
Dragging, pushing, or moving heavy objects		
None	(ref)	(ref)
Mild	1.89 (1.76–2.04)*	1.38 (1.27–1.50)*
Moderate	2.68 (2.45–2.93)*	1.64 (1.49–1.82)*
Severe	3.34 (2.94–3.79)*	2.09 (1.82–2.40)*
Standing posture		
None	(ref)	(ref)
Mild	1.45 (1.32–1.60)*	1.09 (0.98–1.20)
Moderate	2.03 (1.84–2.24)*	1.35 (1.21–1.50)*
Severe	2.56 (2.30–2.84)*	1.79 (1.60–2.01)*
Repetitive hand or arm movements		
None	(ref)	(ref)
Mild	1.47 (1.31–1.64)*	1.23 (1.10–1.38)*
Moderate	2.35 (2.11–2.62)*	1.69 (1.51–1.90)*
Severe	3.08 (2.76–3.44)*	2.04 (1.82–2.30)*

Abbreviations: *LBP* lower back pain

Data presented as odds ratios and 95% confidence intervals

^a^Adjusted for sex, age, occupational type, employment status, working hours, shift work, number of employees, education, income, rest during work time, and vibration exposure

*p-value < 0.001

**p-value < 0.05

The relationship between simultaneous exposure to 2 ergonomic risk factors (Table 1) and prevalence of work-related LBP was analyzed using logistic regression (Tables 4, 5 and 6). Compared to the group with low exposure to 2 risk factors, usually being exposed to 1 risk factor was a relationship with work-related LBP. However, the relationship between high exposure to 2 risk factors and work-related LBP did not increase when comparing exposure to only 1 risk factors. Usually exposed group to ‘fatigue-inducing or painful posture’ (aOR 2.17, 95% CI 2.02–2.34) and high exposed group to both ‘fatigue-inducing or painful posture’ and ‘dragging, pushing, or moving heavy objects’ (aOR 2.00, 95% CI 1.82–2.20) had similar adjusted odd ratio (Table 4). Table 5 (between ‘fatigue-inducing or painful posture’ and ‘repetitive hand or arm movements’) and Table 6 (‘repetitive hand or arm movements’ and ‘dragging, pushing, or moving heavy objects’) had similar result with Table 4.

**Table 4:** Logistic regression analysis for work-related LBP and 2 Ergonomic risk-factors (‘Fatigue-inducing or painful posture’ and ‘Dragging, pushing, or moving heavy objects’)

	Work-related LBP	
Ergonomic risk exposure	Crude	Adjusted^a^
Little exposure to both ‘fatigue-inducing or painful posture’ and ‘dragging, pushing, or moving heavy objects’ (*n* = 24,819)	Reference	Reference
Usually exposed to ‘fatigue-inducing or painful posture’ (*n* = 7920)	3.01 (2.82–3.22)*	2.17 (2.02–2.34)*
Usually exposed to ‘dragging, pushing, or moving heavy objects’ (*n* = 2769)	1.72 (1.54–1.92)*	1.33 (1.19–1.50)*
High exposure to both ‘fatigue-inducing or painful posture’ and ‘dragging, pushing, or moving heavy objects’ (*n* = 5244)	3.04 (2.82–3.28)*	2.00 (1.82–2.20)*

Abbreviations: *LBP* lower back pain

Data presented as odds ratios and 95% confidence intervals

^a^Adjusted for sex, age, occupational type, employment status, working hours, shift work, number of employees, education, income, rest during work time, vibration exposure, ‘lifting or moving people’, ‘standing posture’, ‘repetitive hand or arm movements’

*p-value < 0.001

**Table 5:** Logistic regression analysis for work-related LBP and 2 Ergonomic risk-factors (‘Fatigue-inducing or painful posture’ and ‘Repetitive hand or arm movements’)

	Work-related LBP	
Ergonomic risk exposure	Crude	Adjusted^a^
Little exposure to both ‘fatigue-inducing or painful posture’ and ‘repetitive hand and arm movements’ (*n* = 16,713)	Reference	Reference
Usually exposed to ‘fatigue-inducing or painful posture’ (*n* = 2632)	2.93 (2.63–3.26)*	2.31 (2.06–2.60)*
Usually exposed to ‘repetitive hand or arm movements’ (*n* = 10,875)	1.54 (1.42–1.67)*	1.30 (1.19–1.42)*
High exposure to both ‘fatigue-inducing or painful postur’e and ‘repetitive hand or arm movements’ (n = 10,532)	3.55 (3.31–3.81)*	2.39 (2.19–2.60)*

Abbreviations: *LBP* lower back pain

Data presented as odds ratios and 95% confidence intervals

^a^Adjusted for sex, age, occupational type, employment status, working hours, shift work, number of employees, education, income, rest during work time, vibration exposure, ‘lifting or moving people’, ‘dragging, pushing or moving heavy objects’, ‘standing posture’

*p-value < 0.001

**Table 6:** Logistic regression analysis for work-related LBP and 2 Ergonomic risk-factors (‘Repetitive hand or arm movements’ and ‘Dragging, pushing, or moving heavy objects’)

	Work-related LBP	
Ergonomic risk exposure	Crude	Adjusted^a^
Little exposure to both ‘repetitive hand or arm movements’ and ‘dragging, pushing, or moving heavy objects’ (*n* = 17,547)	Reference	Reference
Usually exposed to ‘repetitive hand or arm movements’ (*n* = 15,192)	2.00 (1.88–2.14)*	1.25 (1.15–1.34)*
Usually exposed to ‘dragging, pushing, or moving heavy objects’ (*n* = 1798)	2.21 (1.95–2.52)*	1.37 (1.19–1.57)*
High exposure to both ‘repetitive hand or arm movements’ and ‘dragging, pushing, or moving heavy objects’ (*n* = 6215)	2.70 (2.50–2.92)*	1.49 (1.39–1.59)**

Abbreviations: *LBP* lower back pain

Data presented as odds ratios and 95% confidence intervals

^a^Adjusted for sex, age, occupational type, employment status, working hours, shift work, number of employees, education, income, rest during work time, vibration exposure, ‘fatigue-inducing or painful posture’, ‘lifting or moving people’, ‘standing posture’

*p-value < 0.001

**p-value < 0.05

## Discussion

This study analyzed relationship between ergonomic risk factors and work-related LBP in Koreans, using large-scale survey data. The prevalence of work-related LBP increased with age, and was higher in blue-collar occupations than in white-collar occupations. Interestingly, the prevalence of work-related LBP was higher in self-employed individuals than in paid workers, and the prevalence was higher in self-employed individuals without employees. This suggests that self-employed individuals in Korea are also exposed to ergonomic risk factors and may be at a greater risk of developing work-related LBP. Symptoms were also more prevalent when there was a smaller number of employees in the participant’s workplace. This may be due to increased workload requirements or greater exposure to ergonomic risk factors in smaller businesses. In the light of these results, small business owners in agriculture, transportation, or restaurants are exposed similarly to ergonomic risk factors as paid workers are. Thus, future studies that investigate ergonomic risk factors in Koreans should include both small business owners and paid workers.

Work-related LBP were more prevalent in those who were unable to take breaks during work than those who could take breaks as required. Although some studies have found that shift work affects musculoskeletal symptoms [26], we did not find a significant relationship in this study. However, this may be attributable to the fact that we only analyzed whether shift work was performed. Further detailed analysis of shift work might have yielded different results. In our study, the prevalence of work- related lower back symptoms tended to increase with increasing severity of exposure to vibration. However, one of longitudinal studies provided no evidence for the relationship between vibration and low back WMSD [9].

Excluding the risk factor of lifting or moving people, the relationship between exposure and work-related LBP was increased with increasing exposure to each risk factors. In previous study, LBP has a strong correlation with moving or pushing objects and whole-body vibration and has a moderate correlation with fatigue-inducing or painful posture and has a low correlation with standing posture, such as static work posture [22]. Fatigue-inducing or painful posture, as stated in the fourth KWCS, refers to posture that occurs when looking back or bending the back. This awkward posture was known to be well correlated with LBP [27] and this was also supported by our findings. In our study, work-related LBP was most strongly associated with ‘fatigue-inducing or painful posture’ risk factor and these awkward postures have been reported to have causal relationship with low back WMSD [9]. There is a theory that suggests that actual internal derangement related to higher mechanical stresses, such as from continually working in an awkward posture, may lead to more disabling forms of LBP. However, the biological plausibility of this theory requires further evidence from additional higher quality studies [28].

Moving people is generally believed to ergonomic risk factor [29–31]. However, interestingly, mild or moderate exposure to ‘lifting or moving people’ group was tended to have less work-related LBP than the exposed groups and only severe exposure group was strongly correlated with work-related lower back symptoms. As a result of our search, such results have not been reported in previous studies. Compared to the daily frequency of moving or lifting objects, the daily frequency of moving or lifting people may be much lower. Also, mildly engaging in moving or lifting people may serve as a strengthening exercise for the body.

Ergonomic risk factors could be seen as potential factors towards the development of work-related LBP and most studies investigated the combined effect of these factors. We performed additional analysis to investigate whether being simultaneously exposed to 2 ergonomic risk factors increases the relationship with work-related lower back symptoms (Tables 4, 5 and 6). When severely exposed to 2 risk factors, the degree of relationship between exposure and work-related LBP was similar to that when only usually exposed to 1 risk factor. Previous epidemiological studies suggest that there is no combined effect of ergonomic risk factors on back pain, however, elbow and hand/wrist have a combined effect [22]. The similar results were found in our study involving a Korean population. Although combined effect of ergonomic risk factors is less likely to be associated with work-related LBP, another study had shown that simultaneous exposure to inadequate posture and wholebody vibration increases the risk of back pain [22, 32]. In the future, it seems that studies related to the combined effects of ergonomic risk factors for other WMSDs (e.g., elbow) besides back WMSD are needed.

This study has some limitations. First, as the KWCS is a cross-sectional study, only relation between symptoms and risk factors could be established, not causal relationships. Second, although surveyors conducted 1:1 interview, ergonomic risk factors and work-related back pain symptoms were self-reported, rendering them vulnerable to response bias. The use of self-reports as a way of evaluating ergonomic risk factors is known to be imprecise and unreliable [33]. Our study was based on questionnaire so that it could be vulnerable misclassification of both exposure and outcome and it could have recall bias. In addition, although it has been previously reported that WRMDs are highly associated with body mass index (BMI) [34], BMI data was not available in the KWCS. Additionally, although vibration exposure could be divided into whole-body vibration, motion sickness, and hand-transmitted vibration [35], the survey did not include this breakdown of vibration types, potentially introducing error. Previous studies have reported that workers who do not wear personal protection devices are more easily exposed to hazardous factors such as noise or vibration [36], but this study could not adjust for the use of personal protection devices. Additionally, we could not adjust for variables such as job stress or psychosocial work environment, which have also been suggested to ergonomic risk factors [37–39].

Despite these limitations, this study was focused on simultaneous exposure to multiple ergonomic risk factors. In addition, large-scale survey data was used and we analyzed not only paid workers, but also self-employment without employee. Our study suggests that self-employed workers should be included in the study of WMSD in the future.

## Conclusions

There was a strong relationship between severe exposure to each ergonomic risk factors (fatigue-inducing or painful posture; lifting or moving people; dragging, pushing, or moving heavy objects; standing posture; repetitive hand or arm movements) and work-related LBP. When exposed to 2 ergonomic risk factors simultaneously, the relationship between exposure and work-related LBP was not stronger than when exposed to only 1 risk factor in our study. Further studies are needed to investigate the combined effect of ergonomic risk factors to other WMSD (e.g., elbow, hand/wrist).

## Abbreviations

BMI: Body mass index
KOSHA: Korea Occupational Safety and Health Agency
KWCS: Korean Working Conditions Survey
LBP: Lower back pain
WMSD: Work-related musculoskeletal disorder
